# Revision of failed-posterior cruciate ligament (PCL) reconstruction due to tibial tunnel misplacement: A case report

**DOI:** 10.1016/j.amsu.2019.10.022

**Published:** 2019-10-30

**Authors:** Andri Maruli Tua Lubis, Mohamad Walid Kuncoro

**Affiliations:** a Department of Orthopaedics and Traumatology, Cipto Mangunkusumo General Hospital, Jakarta, Indonesia; b Department of Orthopaedics and Traumatology, Faculty of Medicine Universitas Indonesia, Jakarta, Indonesia

**Keywords:** PCL reconstruction revision, Tibial tunnel placement

## Abstract

**Introduction:**

Posterior cruciate ligament (PCL) reconstruction failure is a rare condition found. The failure caused by some factors, including improper graft tunnel placement. Although the proper tibial tunnel placement in PCL reconstruction is still controversial, make the tunnel placement anatomically essential to decrease the risk of failure. The use of PCL jig only to guide the direction of tibial tunnel does not always give good results.

**Presentation of case:**

We report a case of 29 year old male with total rupture of ACL and PCL that underwent reconstruction for both ligaments. We found the failure of the PCL graft 2 years after the surgery was related to the tibial tunnel placement which was placed not in proper anatomical site. We performed revision PCL surgery with transseptal portal technique to ensure the tibial tunnel is placed in appropriate position.

**Discussion:**

The cause of failure was associated with misposition of tibial tunnel. The tibial tunnel performed in previous surgery was too anterior than the anatomical foot print. This condition might be caused by surgical technique which depending only on PCL jig to guide the tibial tunnel direction and location. We performed transseptal portal technique get better visualization on the posterior aspect of the knee to achieve the proper direction of tibial tunnel.

**Conclusion:**

The use of PCL jig as the only tools for guiding tibial tunneling should be avoided. Additional tool such as transseptal portal is required to ensure the proper anatomical tibia tunnel in order to achive good PCL graft placement.

## Highlights

• The tibial tunnel placement is important in PCL reconstruction procedure.
• The tibial jig for PCL reconstruction is not always reliable.
• Transseptal view is an important alternative for creating tibial tunnel in PCL reconstruction.

## 1 Introduction

Posterior cruciate ligament (PCL) is a ligament crossing from lateral aspect of medial femoral condyle to posterior aspect of proximal tibia. This structure primarily act to restraint posterior translation of tibia. It also restraint the varus, valgus and external rotation of knee joint [1]. This is an extrasynovial intra-articular structure lined by synovial sheath. The dimensions of PCL was 32–38 mm long and 11 mm cros-sectionally. Based on its function during flexion and extension, it divide into anterolateral and posteromedial bundle, where the anterior one will tight in flexion and lax in extension of the knee whereas the posterior one will tight in extension and lax in extension [1,2].

PCL rupture management is still controversial due to less data established in the literartures. Some modalities of treatment are proposed to solve PCL problems. Conservative management for isolated PCL rupture has been reported to have satisfying results in 80% patients [3]. Some data recommended that conservative management is suitable for grade I and II PCL rupture, whereas grade III PCL injuries with pain and instability as the sympotoms will be failed by conservative way and need surgical treatment to prevent PCL insuficiency and its effect for mechanical load of the knee [3]. Surgical treatment is also addressed for better long-term outcome which cannot be achieved by non-operative management.

Tibial tunnel placement is considered very important in PCL reconstruction. Although the data about proper tibial placement is not well established yet, the anatomical placement is preferred to achieve good functional outcome. Some techniques used to make proper placement for tibial tunnel, PCL jig usually used for guiding the drill of tunnel. Other tools used is image intensifier which is done intraoperatively and also transseptal portal that can show us posterior aspect of tibia clearly. Direct vision to the posterior compartment is important and more favourable. However, the posterior compartment is an area that cannot easily accesible unless using open surgery. Regarding some risks and complications due to open surgery, such as injury of neurovasucular bundle, some techniques using arthroscopy are developed. This report is aimed to discuss about how to place tibial tunnel properly on its anatomical footprint by using transseptal portal [[3], [4], [5]].

This report is based on consensus-based surgical case report guidelines, SCARE criteria [6].

## 2 Case presentation

We report a case of a 29-year-old male patient with chief complaints were pain and instability on his left knee. He got injured when playing futsal with knee twisted externally. He suffered severe pain, swollen and difficult to walk. The magnetic resonance imaging (MRI) examination after the injury revealed ACL and PCL rupture of the left knee. He underwent arthroscopic surgical reconstruction for both ACL and PCL by previous surgeon. Two years after surgery, he felt instability, swollen knee without pain and no history of other trauma during the time after operation. On the physical examination we found posterior sagging and positive posterior drawer test as well as quadriceps active test. Anterior Lachman test for anterior cruciate ligament was negative.

**Fig. 1:**
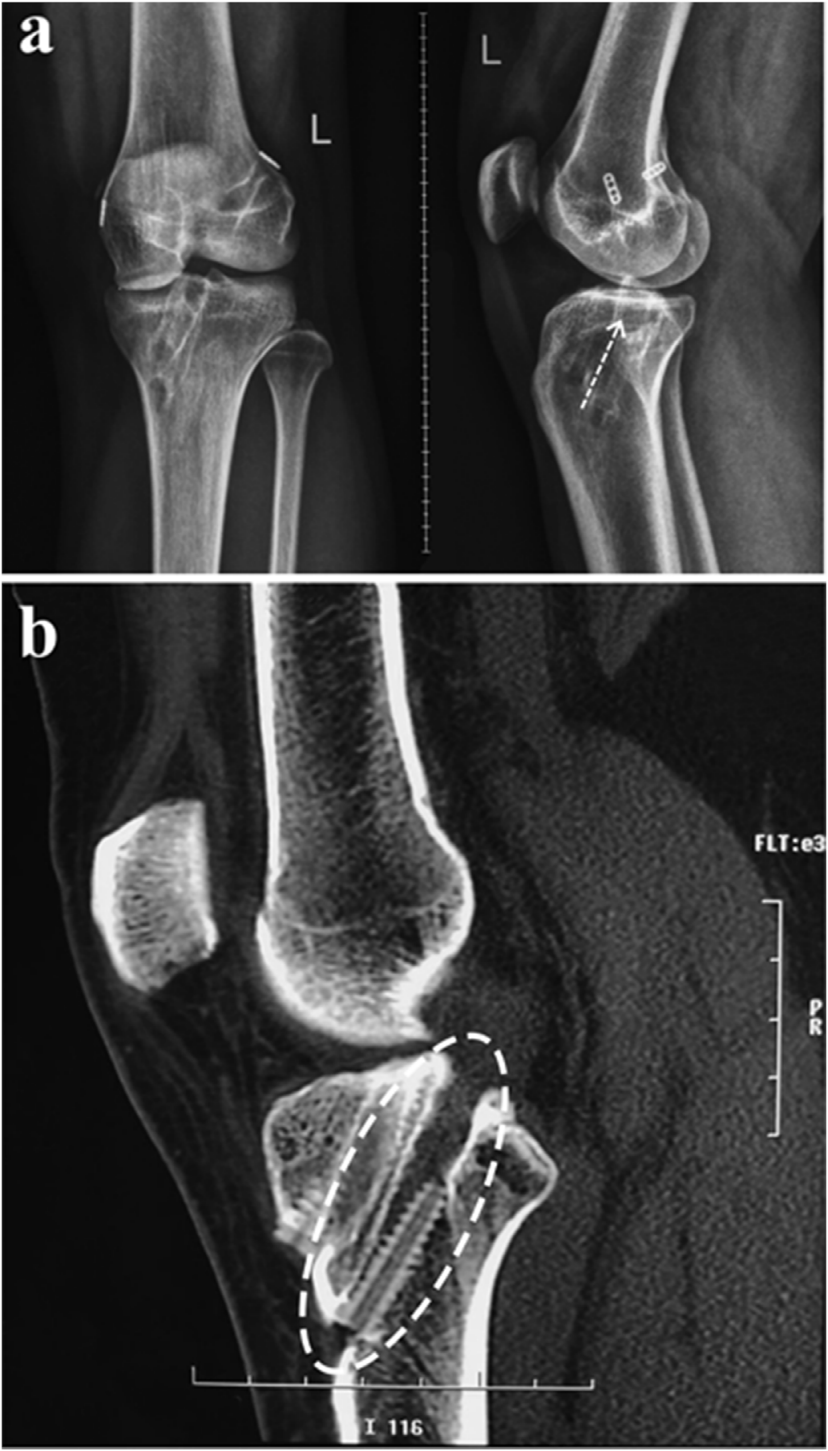
Post-PCL-reconstruction.**(a)** Plain X-ray showed that angel and direction of tibial tunnel was too anterior (white arrow) and **(b)** 3D-CT scan of left knee showed that tibial tunnel for PCL was too anterior than normal footprint (white circle).

On the MRI, we found failure of PCL graft with intact ACL. On the MRI and three dimensional CT scan, with more clear projection for bone structure, it was found that the tibial tunnel placement done in previous surgery was not placed on its anatomical position. The tunnel was placed too anterior to the PCL footprint (Fig. 1).

**Fig. 2:**
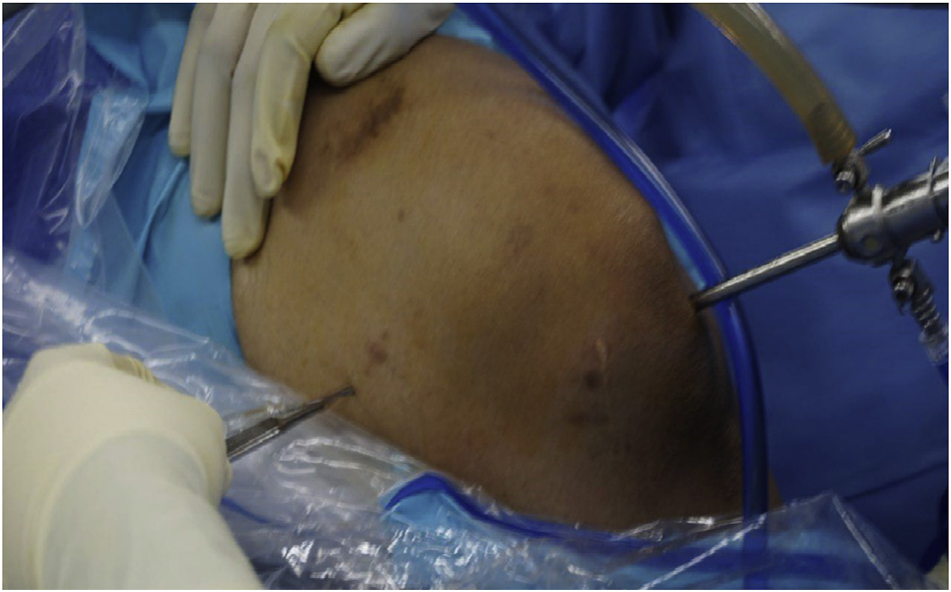
Posteromedial incision of transseptal portal. The site of incision was guided by arthroscopy view.

Subsquently we performed the PCL revision reconstruction surgery. We performed the arthroscopic-assisted reconstruction surgery using transseptal portal approach. We avoided to use only the jig to guide us when tunnelling the tibia instead, we used additional technique to see the posterior aspect of proximal tibia clearly. In this case, we choosed to make a transseptal portal that penetrated from posteromedial side of the knee inside-out to the posterolateral side of the knee (Fig. 2). An incision was made on the posteromedial side of the knee with guidance of arthroscopic view and also transiluminatic arthroscopic light. Blunt obturator with sheath was inserted gently passed through intercondylar notch to posterolateral side of the knee and we made inside-out incision on it.

**Fig. 3:**
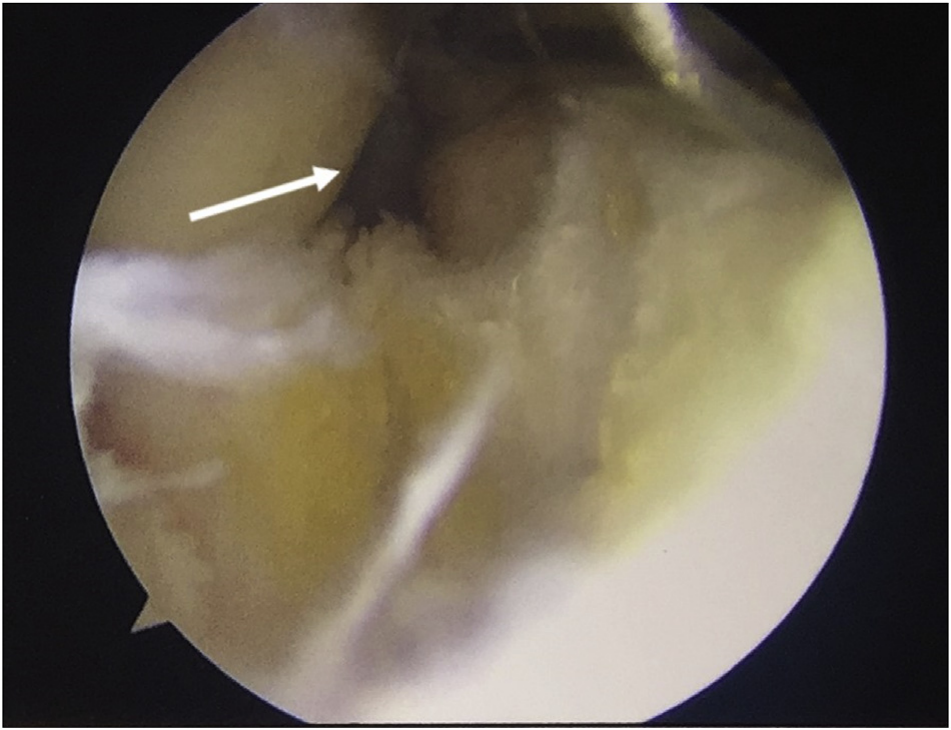
Transeptal portal viewed from posterolateral portal (white arrow).

**Fig. 4:**
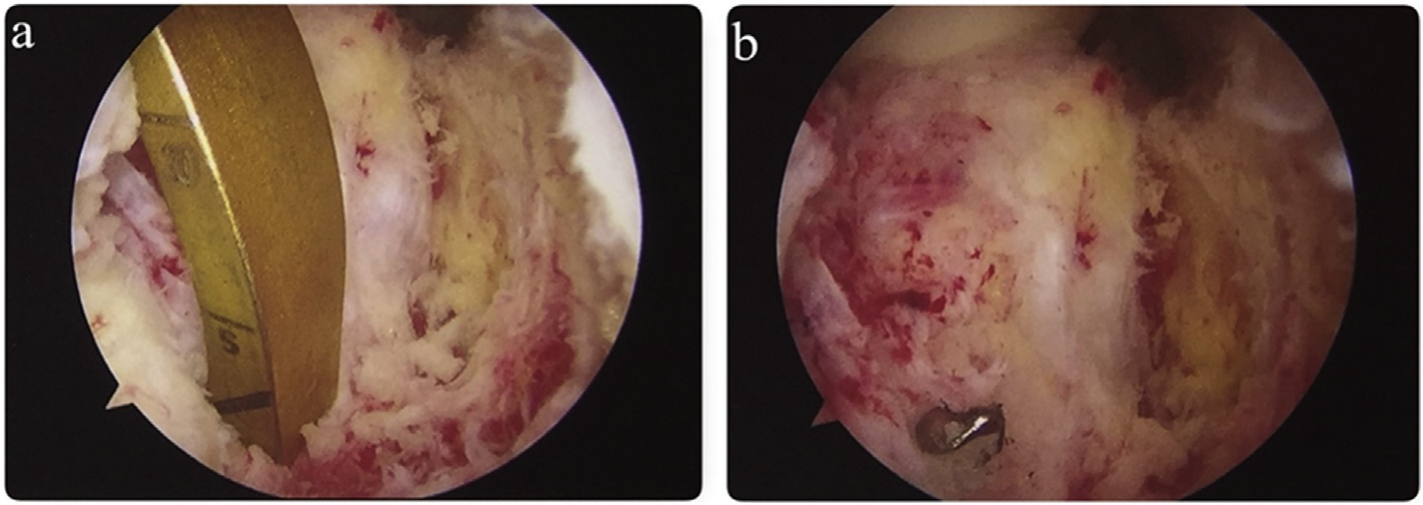
(a) Transseptal portal view of the posterior aspect of the tibia showing that the jig placement was in proper site, and (b) the drill guide was the drill guide pierced the tibia and exits the to posterior aspect right at the end of the jig.

**Fig. 5:**
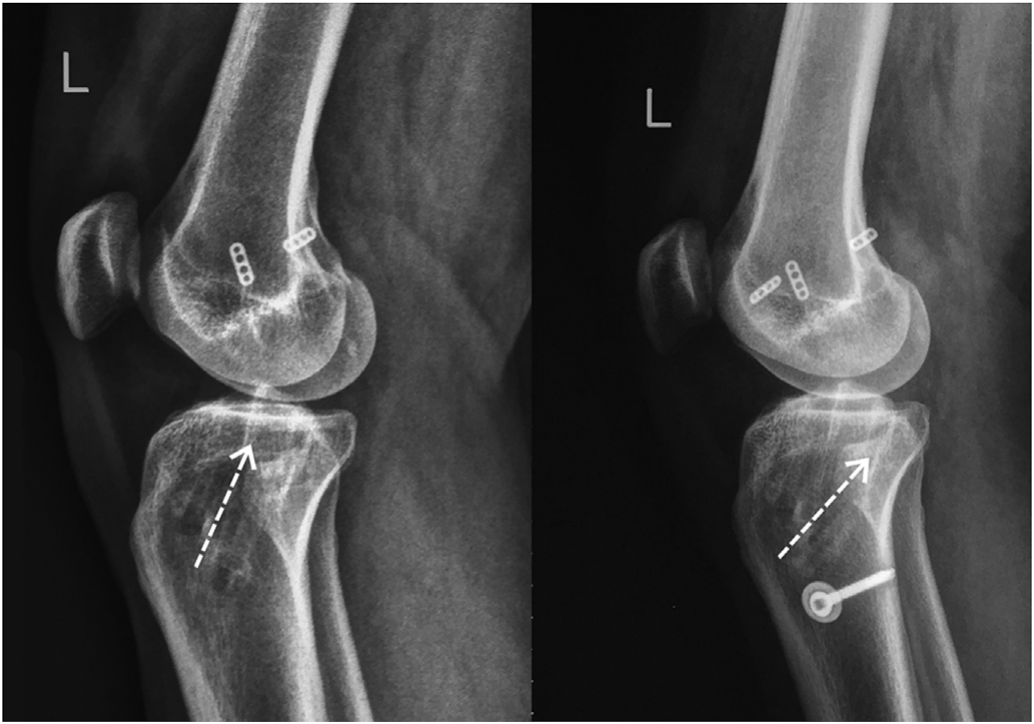
Comparative lateral x-ray before and after revision, tibial tunnel direction was replaced by new tunnel.

During arthroscopy procedure, we found that the PCL was gone with small PCL remnant on femoral site. The ACL was still intact and adequately attached. We performed the reconstruction of PCL using peroneus longus tendon as the graft from the left ankle. When tunneling the tibia, we used jig guide for tibial tunnel placement, we also made a transseptal portal from medial to lateral in order to get better view of posterior aspect of the tibia (Fig. 3 and Fig. 4). We used it as the graft because hamstring tendon was already used in previous surgery. Fig. 5 showed post-operative X-ray of the left knee that tibial tunnel was revised to appropriate site of its footprint. The shadow of two endobuttons on the lmedial femoral condyle was seen because the endobutton of previous surgery was not removed.

## 3 Discussion

Posterior cruciate ligament injury is a rare condition. The epidemiologic data shown PCL injuries representing 1%–44% of all knee ligament injuries. This kind of ligament injury comes primarily from two different setting, i.e. vehicular accident (45%) and sports injuries (40%). A PCL tear occurred as isolated injury in 15.3% and accompanied by other structures in 84.7%, with the most common structure involved is ACL (48.2%) [7,8]. In our case, the patient suffered both ACL and PCL rupture due to sport activity.

The cause of PCL reconstruction failure in this case was due to the improper tibial tunnel placement in the previous surgery, which was too anterior related to PCL anatomical footprint. Our presumption was based on literature stated that malpositioning of tibial tunnel seems to be the most important causes of graft failure [9]. Noyes et al. found in their case series that malposition of either femoral or tibial tunnel contributed in graft failure in second most common causes (33%) after the existed deficiency of LCL and posterolateral ligament as the first common cause (40%) [10]. Johansenn et al. stated that too proximal placement of tibila tunnel leads to decreases ability of the graft to resist posterior translation of the tibia and lead to graft failure subsquently, but this theory is still controversial [11]. On the other hand, Nicodeme et al. in their systematic review about tibial tunnel placement showed there was no significant difference between more anterior or posterior of tibial tunnel placement in anteroposterior laxity [12]. However, a significant difference was found between medial and lateral placement. Nevertheles, based on the absence of other clinical evidence, they recommended that tibial tunnel position for PCL reconstruction should be anatomical [12]. Some surgeons propose to use image intensifier to guide the achievement of good tibial tunnel.

Posterior aspect of the tibia is not an easy area to access arthroscopically. In the past, surgeons worried to damage the neurovascular bundle that lies just behind the posterior capsule if they performed arthroscope insertion posteriorly. This condition consequenced in more difficult of surgical procedure in that compartment [4,5,12].

In this patient, we tried to achieve proper position of tibial tunnel by jig PCL guide and also transseptal portal for better visualization at posterior part of the proximal tibia. In adition to anterolateral and anteromedial portals for arthroscopy, we performed incision and introduced the trochar from posteromedial to the posterolateral side of the knee. These approaches are known as transseptal portal, firstly introduced by Ahn in 2000 to approach posterior compartment of the knee. He stated that this technique was simple and gives a wide visualization to the posterior compartment. Some authors proposed transseptal portal confers several advantages [5]. Ohishi et al. stated that transseptal portal for posterior compartment visualization is less invasive surgical procedure compared to open surgery. Lee et al. showed that there were advantages and disadvantages of transseptal portal procedure [9]. This procedure improves exposure of PCL attachment site on the tibia, preserves maximal amount of remnant PCL, minimalize neurovascular injury and killer-turn effect [13,14].

## 4 Conclusion

In conclusion, PCL reconstruction failure is still a challenge. Appropriate technique should be attempted in order to prevent the failure and achieve good functional outcome for the patient. The use of PCL jig as the only tools for guiding tibial tunneling should be avoided. Additional tools like image intensifier or transseptal portal are required to ensure the proper tibial tunnel anatomically in order to achive good PCL graft placement.

## Provenance and peer review

Not commissioned, editor reviewed.

## Ethical approval

This is a case report; therefore it did not require ethical approval from ethics committee. However, we have got permission from the patient to publish his data.

## Author contribution

Andri Lubis contributed in performing the surgical procedure, data collection, data analysis and writing the paper.

Mohamad Walid Kuncoro contributed in data collection, data analysis and writing the paper.

## Consent

We have written and signed informed consent obtained from the patient to publish this case report and accompanying imagesConflict of Interest.

## Funding

No sponsorship for this case report.

## Registration of research studies

This is a case report; therefore, we did not register this work in clinical trial registry.

1. Name of the registry: N/A.

2. Unique Identifying number or registration ID: N/A.

3. Hyperlink to the registration (must be publicly accessible): N/A.

## Guarantor

The Guarantor is Andri M.T. Lubis, M.D., Ph.D.

## Declaration of competing interest

The authors declare that there is no conflict of interest regarding publication of this paper.
